# Proteinuria and hematuria are associated with acute kidney injury and mortality in critically ill patients: a retrospective observational study

**DOI:** 10.1186/1471-2369-15-93

**Published:** 2014-06-18

**Authors:** Seung Seok Han, Shin Young Ahn, Jiwon Ryu, Seon Ha Baek, Ho Jun Chin, Ki Young Na, Dong-Wan Chae, Sejoong Kim

**Affiliations:** 1 Department of Internal Medicine, Seoul National University College of Medicine, Seoul, Korea; 2 Department of Internal Medicine, Seoul National University Bundang Hospital, Seongnam-si, Gyeonggi-do, Korea; 3 Department of Internal Medicine, Hallym University College of Medicine, Chuncheon-si, Gangwon-do, Korea; 4 Department of Biomedical Engineering, University of Michigan, Ann Arbor, USA

**Keywords:** Acute kidney injury, Hematuria, Intensive care unit, Mortality, Proteinuria

## Abstract

**Background:**

Proteinuria and hematuria are both important health issues; however, the nature of the association between these findings and acute kidney injury (AKI) or mortality remains unresolved in critically ill patients.

**Methods:**

Proteinuria and hematuria were measured by a dipstick test and scored using a scale ranging from a negative result to 3+ in 1883 patients admitted to the intensive care unit. AKI was defined according to the Kidney Disease: Improving Global Outcomes (KDIGO) guidelines. The odds ratios (ORs) for AKI and 3-year mortality were calculated after adjustment for multiple covariates according to the degree of proteinuria or hematuria. For evaluating the synergistic effect on mortality among proteinuria, hematuria, and AKI, the relative excess risk due to interaction (RERI) was used.

**Results:**

Proteinuria and hematuria increased the ORs for AKI: the ORs of proteinuria were 1.66 (+/−), 1.86 (1+), 2.18 (2+), and 4.74 (3+) compared with non-proteinuria; the ORs of hematuria were 1.31 (+/−), 1.58 (1+), 2.63 (2+), and 2.52 (3+) compared with non-hematuria. The correlations between the mortality risk and proteinuria or hematuria were all significant and graded (*P*trend < 0.001). There was a relative excess risk of mortality when both AKI and proteinuria or hematuria were considered together: the synergy indexes were 1.30 and 1.23 for proteinuria and hematuria, respectively.

**Conclusions:**

Proteinuria and hematuria are associated with the risks of AKI and mortality in critically ill patients. Additionally, these findings had a synergistic effect with AKI on mortality.

## Background

Acute kidney injury (AKI) is a major focus of study in nephrology because AKI is related to an increase in morbidity and mortality [1,2]. Although therapy for AKI has improved in recent years, AKI is still highly prevalent, especially in critically ill patients in the intensive care unit (ICU) [3]. AKI in the ICU has extremely high mortality rates, reaching 80% [4]; this rate has remained relatively unchanged despite improved therapies [5]. For these reasons, the detection and management of factors related to AKI are important concerns for clinicians in managing AKI patients.

Urinary abnormalities include proteinuria, hematuria, oliguria, frequency, and polyuria. These features can be encountered in various clinical settings; oliguria, one of the possible findings, comprises 50% of AKI cases [6] and is included in the criteria for defining AKI [7]. However, other urinary features such as proteinuria and hematuria have not been thoroughly considered in connection with AKI. Proteinuria and hematuria can be easily screened by a dipstick test. These are increasingly recognized as important markers of disease that carry risks for end-stage renal disease [8]. Previous studies have described the association between AKI and proteinuria or hematuria in certain clinical settings, such as outpatient [9], cardiac surgery [10,11], burn [12], immunoglobulin A nephropathy [13], and warfarin-induced nephropathy [14]. However, these studies have not considered proteinuria and hematuria together or the ICU patients who have a high risk of AKI and mortality. The combined study of proteinuria, hematuria, and AKI may be necessary to improve the predictability of outcomes, but there are also no studies that address this issue.

Herein, we aim to verify whether proteinuria or hematuria increase the risks of AKI or mortality in a large cohort of ICU patients. Furthermore, we examine the synergistic effect of proteinuria, hematuria, and AKI for predicting mortality.

## Methods

### Participants and data collection

The study protocol complies with the Declaration of Helsinki and had ethics approval from the institutional review board at the Seoul National University Bundang Hospital (no. B-1304/200-108). A total of 2823 patients were consecutively recruited from June 2004 through June 2010, who were admitted to the ICU at the Seoul National University Bundang Hospital, Gyeonggi-do, Korea. The patients were followed up until June 30, 2010. We excluded patients younger than 18 years old (n = 49) and patients previously diagnosed with end stage renal disease on dialysis (n = 94). The patients for whom data were missing, including serum creatinine, urine output, or dipstick test results, were also excluded (n = 250). Lastly, the patients with a daily urine output of less than 50 mL/day or anuria were excluded (n = 23). If the patients were admitted more than once to the ICU, only the first admission was counted as the single case. Consequently, 1883 patients were reviewed retrospectively using electronic medical records. The need for informed consent was waived because of non-interventional study design based on routinely collected data.

Clinical parameters, such as age, sex, weight, systolic/diastolic blood pressure, primary diagnosis, underlying chronic kidney disease, diabetes mellitus, history of malignancy, the need for mechanical ventilation, and the use of vasoactive drugs, were recorded. The primary diagnoses included cardiovascular disease, sepsis, surgical admission, and others. The Acute Physiology and Chronic Health Evaluation (APACHE) ІІ score was used to assess illness severity [15]. Changes in serum creatinine and urine output after ICU admission were measured, and the urine output data were recorded hourly. A dipstick test was used to score the degrees of proteinuria and hematuria using a scale ranging from a negative result to +3 using an automated urine chemistry analyzer (CLINITEK Novus Analyzer, Siemens, Erlangen, Germany). There were no missing data for any of the variables.

The risk of AKI was determined from admission to 15 days in the ICU. For the definition and staging of AKI, both the serum creatinine and the urine output criteria were used according to the guideline proposed by the Kidney Disease: Improving Global Outcomes (KDIGO) [7]. The 3-year mortality from all causes was also considered to be the primary outcome. The mortality data were obtained from the national database of Statistics Korea.

### Statistical analysis

All of the analyses and calculations were performed using STATA (STATA version 12.0, StataCorp LP, College Station, Texas, USA). The data are presented as means ± standard deviation (SD) for continuous variables and as proportions for categorical variables. Comparisons were measured using the chi-squared test for categorical variables (e.g., AKI stages) among the proteinuria and hematuria groups. Mortality curves were drawn using the Kaplan-Meier method. A logistic regression analysis was used to examine the risk of AKI or 3-year mortality according to the presence of proteinuria or hematuria. The effects of the logistic regression model are shown as odds ratios (ORs) and 95% confidence intervals (CIs). For this analysis, ORs were adjusted for multiple covariates, such as age, sex, body weight, primary diagnosis, underlying chronic kidney disease, diabetes mellitus, history of malignancy, the need for mechanical ventilation, the use of vasoactive drugs, and APACHE ІІ score. To evaluate the synergistic effect on mortality of proteinuria, hematuria, and AKI, the relative excess risk due to interaction (RERI) was used [16]. The RERI is an approach to estimate the additive interaction of two variables on an odds ratio scale. From the RERI method, we present three scales: RERI (part of the total effect that is due to interaction), AP (proportion of the combined effect that is due to interaction), and synergy index (ratio between combined effect and individual effects). Positive results for RERI and AP and a value greater than 1 for the synergy index means a positive interaction or more than additivity between variables. Additionally, the discrimination of predicting mortality between AKI and proteinuria or hematuria was assessed by calculating the receiver operating characteristic (ROC) curve and the area under the curve (AUC). The comparison of ROC curves was tested using a method described by DeLong ER et al. [17]. A *P* value of less than 0.05 was considered significant.

## Results

### Baseline characteristics

Among a total of 1883 subjects, the mean age was 68 years old (Table 1). All of the subjects were of Asian descent. Most of the patients were admitted to the ICU because of medical problems (n = 1848) rather than surgical problems (n = 35). More specifically, 580 patients (30.8%) were admitted to the ICU because of cardiovascular disease. Sepsis was the cause of admission for 86 patients (4.6%). The proportion of subjects with trace levels or higher for proteinuria was 67.4% and for hematuria was 77.5%. The mean APACHE ІІ score was 18.4. The median length of stay in the hospital was 22 days (IQR, 11 to 45 days). The study subjects were followed for a median duration of 480 days (IQR, 37 to 1797 days).

**Table 1:** Baseline characteristics and laboratory findings of the patients at the time of admission to the intensive care unit

	**Total (n = 1883)**
Age (years)	67.8 ± 15.88
Male sex (%)	59.9
Body weight (kg)	58.0 ± 12.41
Primary diagnosis (%)	
Cardiovascular disease	30.8
Sepsis	4.6
Surgical emergency	1.9
Others	62.8
Underlying chronic kidney disease (%)	8.7
Diabetes mellitus (%)	12.2
History of malignancy (%)	15.3
Need for mechanical ventilation (%)	69.6
Use of vasoactive drugs (%)	51.4
Systolic blood pressure (mmHg)	130.2 ± 31.52
Diastolic blood pressure (mmHg)	73.0 ± 20.82
Serum creatinine (mg/dL)*	1.0 (0.8 to 1.5)
Proteinuria (%)	
-	32.6
+/−	15.4
1+	26.7
2+	19.3
3+	6.0
Hematuria (%)	
-	22.5
+/−	12.9
1+	12.5
2+	19.3
3+	32.8
APACHE ІІ score	18.4 ± 8.04
Length of stay in hospital (days)*	22 (11 to 45)

*Data are expressed as the median (interquartile range (IQR)) when the distribution of data was skewed.

APACHE, Acute Physiology and Chronic Health Evaluation.

### Risk of acute kidney injury according to proteinuria and hematuria

A total of 78.7% of the subjects had AKI within 15 days after admission to the ICU. 74.7% of total AKI cases were determined at the time of ICU admission, and other cases (25.3%) developed from 2 days to 15 days of admission. Each AKI case was diagnosed by serum creatinine criterion alone (68.1%), urine output criterion alone (2.8%), or both (29.1%). The proportion in each AKI stage was as follows: stage 1, 47.1%; stage 2, 29.7%; and stage 3, 23.2%. Among stage 3 of AKI, 132 subjects (38.4%) received renal replacement therapy. Table 2 shows the risk of AKI according to the presence of proteinuria and hematuria. The risk of AKI gradually increased from the trace level of proteinuria. For hematuria, the risk of AKI increased from the first positive level. The correlations of AKI risk with proteinuria or hematuria remained consistent, irrespective of adjustments made to multiple variables. When patients with both proteinuria (trace or more) and hematuria (1+ or more) (n = 966) were compared with patients without both markers (n = 363), the adjusted HR for AKI was 2.86 (2.102–3.901) (*P* < 0.001). After excluding the AKI cases with onset at day 0, proteinuria and hematuria groups had greater AKI risks (with onset on day 1 to 15) compared with the groups without proteinuria or hematuria as following adjusted HRs: proteinuria [+/−, 1.69 (1.088–2.624) (*P* = 0.020); 1+, 1.49 (1.003–2.212) (*P* = 0.047); 2+, 1.58 (0.991–2.514) (*P* = 0.053); 3+, 2.53 (0.987–6.463) (*P* = 0.052); *P*trend = 0.037]; hematuria [+/−, 1.02 (0.621–1.660) (*P* = 0.951); 1+, 1.47 (0.901–2.409) (*P* = 0.122); 2+, 2.10 (1.315–3.356) (*P* = 0.002); 3+, 1.65 (1.076–2.528) (*P* = 0.022); *P*trend = 0.010].

**Table 2:** Odds ratios for acute kidney injury according to proteinuria and hematuria

	**Univariate**		Multivariate*	
	**OR (95% CI)**	***P***	**OR (95% CI)**	***P***
Proteinuria		< 0.001^†^		< 0.001^†^
- (n = 614)	1 (Reference)		1 (Reference)	
+/− (n = 290)	1.99 (1.426–2.777)	< 0.001	1.66 (1.160–2.385)	0.006
1+ (n = 503)	2.59 (1.938–3.468)	< 0.001	1.86 (1.362–2.551)	< 0.001
2+ (n = 363)	3.04 (2.166–4.278)	< 0.001	2.18 (1.511–3.135)	< 0.001
3+ (n = 113)	6.53 (3.121–13.663)	< 0.001	4.74 (2.199–10.221)	< 0.001
Hematuria		< 0.001^†^		< 0.001^†^
- (n = 423)	1 (Reference)		1 (Reference)	
+/− (n = 243)	1.25 (0.884–1.761)	0.208	1.31 (0.895–1.906)	0.167
1+ (n = 236)	1.63 (1.132–2.341)	0.009	1.58 (1.063–2.349)	0.024
2+ (n = 363)	2.83 (1.989–4.029)	<0.001	2.63 (1.796–3.853)	<0.001
3+ (n = 618)	3.15 (2.315–4.273)	<0.001	2.52 (1.808–3.523)	<0.001

*Adjusted for age, sex, body weight, primary diagnosis, underlying chronic kidney disease, diabetes mellitus, history of malignancy, the need for mechanical ventilation, the use of vasoactive drugs, and APACHE ІІ score.

^†^*P* for trend.

OR, odds ratio; CI, confidence interval.

We further evaluated whether the correlations with proteinuria or hematuria were significant when only one of two criteria (serum creatinine criterion and urine output criterion) was used for identifying AKI. Although the AKI cases were divided by two criteria, proteinuria had greater adjusted ORs for AKI compared with the group without proteinuria as follows; by serum creatinine criterion [+/−, 1.49 (1.054–2.115) (*P* = 0.024); 1+, 1.67 (1.236–2.262) (*P* = 0.001); 2+, 2.01 (1.417–2.860) (*P* < 0.001); 3+, 2.90 (1.534–5.479) (*P* = 0.001); *P*trend < 0.001]; by urine output criterion [+/−, 1.49 (1.028–2.145) (*P* = 0.035); 1+, 1.96 (1.437–2.667) (*P* < 0.001); 2+, 2.07 (1.481–2.882) (*P* < 0.001); 3+, 5.26 (3.310–8.355) (*P* < 0.001); *P*trend < 0.001]. When the patients were compared among the hematuria and non-hematuria groups, the associations with AKI were also significant according to each criterion; by serum creatinine criterion [+/−, 1.32 (0.914–1.918) (*P* = 0.137); 1+, 1.63 (1.105–2.403) (*P* = 0.014); 2+, 2.62 (1.808–3.784) (*P* < 0.001); 3+, 2.29 (1.663–3.150) (*P* < 0.001); *P*trend < 0.001]; by urine output criterion [+/−, 0.97 (0.639–1.478) (*P* = 0.895); 1+, 1.09 (0.717–1.641) (*P* = 0.701); 2+, 1.18 (0.823–1.684) (*P* = 0.372); 3+, 1.82 (1.333–2.479) (*P* < 0.001)].

**Figure 1:**
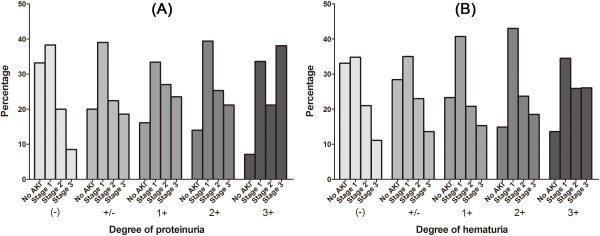
Proportions of acute kidney injury stages in each group: proteinuria (A) and hematuria (B).

The AKI stages in each degree of proteinuria or hematuria are shown in Figure 1. When compared with the group without proteinuria or hematuria, the groups with proteinuria or hematuria demonstrated significant differences in AKI stages; the presence of proteinuria or hematuria led to a tendency toward higher AKI stages than the absence of proteinuria or hematuria (all *P*s < 0.05). The worst case of AKI (stage ІІІ) developed more strongly in the groups with proteinuria or hematuria with the following adjusted ORs (95% CI); proteinuria groups vs. non-proteinuria group [+/−, 2.19 (1.423–3.364) (*P* < 0.001); 1+, 2.75 (1.902–3.970) (*P* < 0.001); 2+, 2.44 (1.637–3.629) (*P* < 0.001); 3+, 6.48 (3.900–10.754) (*P* < 0.001)]; hematuria groups vs. non-hematuria group [+/−, 1.42 (0.867–2.338) (*P* = 0.162); 1+, 1.54 (0.946–2.516) (*P* = 0.082); 2+, 1.78 (1.166–2.727) (*P* = 0.007); 3+, 2.59 (1.784–3.759) (*P* < 0.001)].

### Impact of proteinuria and hematuria on mortality

Throughout the follow-up period, 1270 (67.4%) of all ICU patients died, and the mortality rate was 69.9 deaths per 100000 patient-days. As shown in Table 3, the presence of proteinuria or hematuria worsened the mortality, irrespective of the effects of covariates. Although the AKI was further adjusted, the overall correlations between mortality and proteinuria or hematuria were significant as follows: ORs (95% CI) in proteinuria groups vs. non-proteinuria group [+/−, 1.48 (1.054–2.066) (*P* = 0.023); 1+, 1.71 (1.280–2.273) (*P* < 0.001); 2+, 1.85 (1.347–2.540) (*P* < 0.001); 3+, 1.66 (1.009–2.733) (*P* = 0.046)]; in the hematuria groups vs. non-hematuria group [+/−, 1.13 (0.773–1.652) (*P* = 0.528); 1+, 1.27 (0.866–1.847) (*P* = 0.224); 2+, 1.36 (0.968–1.920) (*P* = 0.076); 3+, 1.46 (1.078–1.988) (*P* = 0.015)]. The trends in the correlations of mortality with proteinuria (*P*trend < 0.001) and hematuria (*P*trend = 0.010) were also significant after further adjustment of AKI. When patients with both proteinuria (trace or more) and hematuria (1+ or more) were compared with patients without both markers, the adjusted HR for mortality was 2.07 (1.545–2.772) (*P* < 0.001).

**Table 3:** Odds ratios for 3-year mortality according to proteinuria and hematuria

	**Univariate**		Multivariate*	
	**OR (95% CI)**	***P***	**OR (95% CI)**	***P***
Proteinuria		< 0.001^†^		< 0.001^†^
- (n = 614)	1 (Reference)		1 (Reference)	
+/− (n = 290)	1.78 (1.340–2.364)	< 0.001	1.59 (1.140–2.213)	0.006
1+ (n = 503)	2.23 (1.750–2.845)	< 0.001	1.86 (1.403–2.472)	< 0.001
2+ (n = 363)	2.49 (1.898–3.268)	< 0.001	2.09 (1.524–2.854)	< 0.001
3+ (n = 113)	2.14 (1.407–3.239)	< 0.001	2.04 (1.239–3.343)	0.005
Hematuria		< 0.001^†^		< 0.001^†^
- (n = 423)	1 (Reference)		1 (Reference)	
+/− (n = 243)	1.08 (0.785–1.477)	0.646	1.17 (0.808–1.703)	0.401
1+ (n = 236)	1.32 (0.955–1.814)	0.094	1.34 (0.921–1.944)	0126
2+ (n = 363)	1.67 (1.257–2.227)	< 0.001	1.55 (1.104–2.163)	0.011
3+ (n = 618)	1.76 (1.370–2.268)	< 0.001	1.65 (1.225–2.232)	0.001

*Adjusted for age, sex, body weight, primary diagnosis, underlying chronic kidney disease, diabetes mellitus, history of malignancy, the need for mechanical ventilation, the use of vasoactive drugs, and APACHE ІІ score.

^†^*P* for trend.

OR, odds ratio; CI, confidence interval.

**Figure 2:**
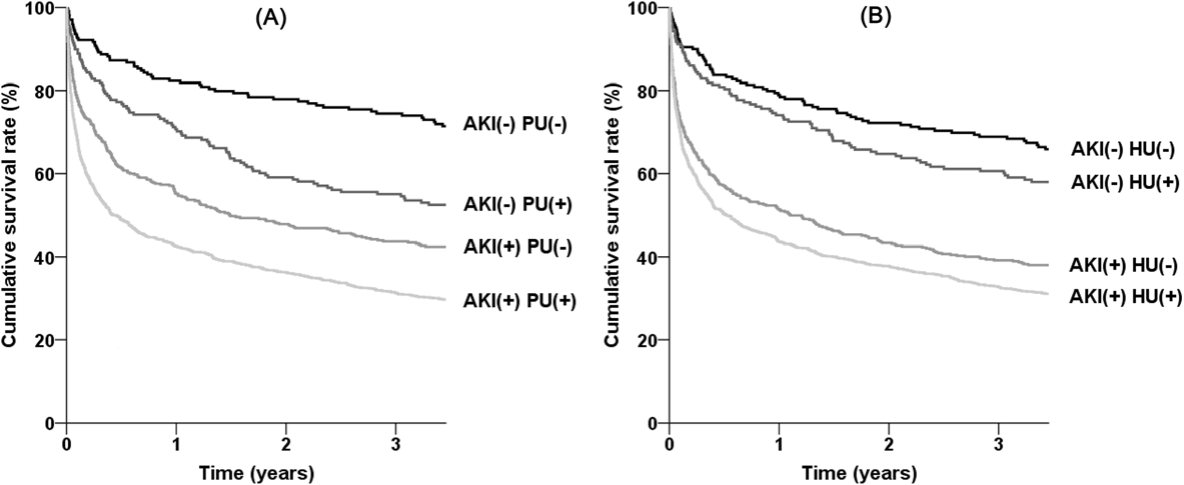
Kaplan-Meier survival curves according to the presence of AKI and proteinuria (A) or hematuria (B).

There was a trend of interaction between AKI and proteinuria for mortality (*P* for interaction = 0.089) or between AKI and hematuria (*P* for interaction = 0.077). We evaluated whether there was a synergistic effect for mortality between AKI and proteinuria or hematuria using the RERI method. Because the outcomes increased above the level of non-proteinuria or trace levels of hematuria, the subjects were divided into two groups as follows: non-proteinuria vs. trace or more for proteinuria; non-hematuria and trace vs. 1+ or more for hematuria. Tables 4 and 5 show the RERI results between AKI and proteinuria or hematuria, respectively. For both proteinuria and hematuria, the RERI and AP scales were positive, and the synergy indexes were greater than 1; this means there was a relative excess risk of mortality when both AKI and proteinuria or hematuria were considered together. The presence of proteinuria or hematuria further separated the AKI-considered survival curves of ICU patients (Figure 2). There was no interaction for AKI or mortality between proteinuria and hematuria (*P* = 0.962).

**Table 4:** Interaction between acute kidney injury and proteinuria on the risk of mortality

	**No proteinuria**		**Proteinuria**		
	**N with/without outcome**	**OR (95% CI)**	**N with/without outcome**	**OR (95% CI)**	**OR for proteinuria within strata of AKI status**
No AKI	52/152	1 (Reference)	89/109	2.39 (1.566–3.637)*	2.39 (1.566–3.637)*
AKI	231/179	3.77 (2.604–5.464)*	736/335	6.42 (4.569–9.026)*	1.70 (1.347–2.151)*
OR for AKI within strata of proteinuria status		3.77 (2.604–5.464)*		2.69 (1.977–3.663)*	

Measure of interaction on additive scale: RERI (95% CI) = 1.26 (−0.153–2.556); AP (95% CI) = 0.20 (−0.230–0.356); SI (95% CI) = 1.30 (0.974–1.750).

*P < 0.001.

OR, odds ratio; CI, confidence interval; AKI, acute kidney injury; RERI, relative excess risk due to interaction; AP, attributable proportion; SI, synergy index.

**Table 5:** Interaction between acute kidney injury and hematuria on the risk of mortality

	**No hematuria**		**Hematuria**		
	**N with/without outcome**	**OR (95% CI)**	**N with/without outcome**	**OR (95% CI)**	**OR for proteinuria within strata of AKI status**
No AKI	65/144	1 (Reference)	76/117	1.44 (0.954–2.171)	1.44 (0.954–2.171)
AKI	278/179	3.44 ( 2.430–4.872)^†^	689/335	4.56 (3.306–6.279)^†^	1.32 (1.054–1.665)*
OR for AKI within strata of hematuria status		3.44 ( 2.430–4.872)^†^		3.17 (2.306–4.347)^†^	

Measure of interaction on additive scale: RERI (95% CI) = 0.68 (−0.543–1.665); AP (95% CI) = 0.15 (−0.118–0.347); SI (95% CI) = 1.23 (0.864–1.853).

*P < 0.05; ^†^*P* < 0.001.

OR, odds ratio; CI, confidence interval; AKI, acute kidney injury; RERI, relative excess risk due to interaction; AP, attributable proportion; SI, synergy index.

### ROC curves for the prediction of acute kidney injury and mortality

We evaluated the ROC curves in proteinuria and hematuria for the prediction of AKI with following AUCs; proteinuria, 0.639 (0.609–0.669); hematuria, 0.630 (0.599–0.661); and proteinuria + hematuria, 0.648 (0.618–0.678). All of AUCs were significantly larger than the reference line (all *P*s < 0.001). We further evaluated whether the predictability of mortality by AKI is improved by the additional consideration of proteinuria and hematuria using AUC comparisons. The AUC (95% CI) of the ROC curve for mortality was 0.605 (0.585–0.624) in the AKI model. The AUCs (95% CI) for mortality in each proteinuria and hematuria model were 0.597 (0.572–0.623) and 0.565 (0.539–0.591), respectively. The AUCs for mortality in the combined models of AKI and urine findings were significantly higher than the AUC calculated using AKI only: AKI + proteinuria, 0.646 (0.620–0.671) (*P* < 0.001); AKI + hematuria, 0.627 (0.602–0.653) (*P* = 0.011); and AKI + proteinuria + hematuria, 0.647 (0.621–0.673) (*P* < 0.001).

## Discussion

The mortality among ICU patients is extremely high, and AKI worsens this outcome. For predicting and managing the mortality attributable to AKI, several markers have been considered. Among them, proteinuria and hematuria are easily examined by a simple dipstick test, but these findings may be paradoxically overlooked despite the inexpensive and easily accessible examination and the intrinsic features of the test. The present study first examines the correlation between AKI and proteinuria or hematuria in ICU patients. Both proteinuria and hematuria predicted the prevalence of AKI. This was consistent regardless of whether AKI was defined by the onset or the criteria. The severity of AKI generally described by stages was higher in the high-degree of proteinuria or hematuria group than in the low-degree group. Both proteinuria and hematuria had a significant association with mortality and additionally had a synergistic effect with AKI. Lastly, the predictability of mortality in the AKI model was improved by considering proteinuria, hematuria, or both.

Proteinuria has been regarded as being important, especially in the field of chronic kidney disease [18]. This is mainly because proteinuria has an impact on mortality, with an effect on mortality similar to smoking [19]. However, the relationship between proteinuria and AKI is not fully established, especially in ICU patients. Previous data on a large cohort of outpatients showed an additive role of proteinuria in predicting the risk of AKI admission [9], although the baseline characteristics were relatively robust (AKI prevalence < 1%). Other cohorts of patients with cardiac surgery and burns, conditions that carry a high risk of AKI, also supported the association between proteinuria and AKI risk [10-12], although those studies did not cover a heterogeneous population of critically ill patients. The present study, which included a large cohort of critically ill patients, builds on the previous study results with respect to the significant correlation between proteinuria and the risk of AKI. Furthermore, it is intriguing that proteinuria and AKI had a synergistic effect on mortality. This means that the risk of mortality is greater than the simple additive risk when AKI and proteinuria are considered together.

Hematuria is an important pathologic finding and thus is included in the criteria for chronic kidney disease [7]. However, its presence is not usually mentioned in large epidemiological studies when compared with proteinuria; most studies in the field of AKI do not focus on the role of hematuria. A few studies described hematuria-associated AKI in certain clinical settings such as immunoglobulin A nephropathy or warfarin-induced nephropathy [13,14], but it is difficult to apply these data directly to subjects with a high risk of AKI. For the first time, the present study demonstrates the associations of hematuria with both AKI and mortality in ICU patients. The effect size of hematuria seemed to be small compared with that of proteinuria, but the effects of hematuria (1+ or more) on AKI and mortality were independent of proteinuria with the following adjusted ORs in the hematuria group compared with the non-hematuria group: AKI, 1.88 (1.476–2.386) (*P* < 0.001); mortality, 1.27 (1.031–1.555) (*P* = 0.024). Furthermore, the predictability of mortality in the AKI model was improved by the addition of hematuria. Based on the present results, further clinical and experimental studies are needed to delineate the role of hematuria in AKI.

Proteinuria and hematuria may simply be markers of underlying kidney disease or other organ status [18,20]. Precisely, urinary abnormalities are shown in more than half of patients with acute tubular necrosis, which is the most common cause of AKI in the ICU [21-23]. Accordingly, these findings may be associated with AKI or mortality via acute tubular necrosis or other baseline conditions. However, in the present study, the correlations of proteinuria or hematuria with outcomes were significant despite adjusting several baseline conditions. Furthermore, both proteinuria and hematuria had an association with the AKI cases with late onset. Investigations on the independent role of proteinuria or hematuria in kidney damage and other types of organ damage are ongoing. It is known that proteinuria induces signals for tubulointerstitial inflammation and activates fibrogenic pathways as a postulated mechanism of kidney damage [24]. Proteinuria is also associated with endothelial dysfunction, which covers cardiovascular mortality [25]. Furthermore, other mechanisms affecting the overall outcome of proteinuria may include an increased risk of infection, thrombotic disease, or malnutrition [26], although thorough investigations have not been conducted. The subjects with hematuria are at risk of kidney damage because hemoglobin, heme, iron, or other molecules released from red blood cells are toxic to the kidney tubular cells and thus induce inflammatory cascades [27,28]. It is intriguing that the cessation of hematuria is related to the recovery of kidney function [13], although this is known only in immunoglobulin A nephropathy, and there are no agents for this.

It is intriguing that hematuria was independently associated with high mortality risk in the ICU patients. There are no observational studies or commentaries on the direct correlation between hematuria and mortality. However, it is known that hematuria increases the risk of AKI or end-stage renal disease development [22,29]. Based on the evidence, we could propose only the following mechanism: hematuria is a marker of kidney injury and thus has a relationship with mortality via progressed (or severe) kidney damage.

Although our results are informative, this study has some limitations. First, the ICU design of the study limits the applicability of our conclusions to other settings, despite the abundance and detail of the dataset. Furthermore, like all observational studies, the present study does not prove causality. Second, the measurement of proteinuria and hematuria by the dipstick test only is a limitation because of the relatively low precision and thus misclassification bias. However, such a bias would have supported the null hypothesis, so the true correlations are most likely stronger than the present study results. Furthermore, the dipstick test is an inexpensive and more accessible examination compared with a quantitative urine test, which has implications in clinical practice. Third, we did not separate preexisting proteinuria and hematuria from newly discovered cases after admission because all the admissions to the ICU were not scheduled. This issue is the same when most patients who are admitted to the ICU do not have the baseline serum creatinine required for defining AKI in principle. However, this condition may better fit the real clinical practice. Further studies addressing these limitations will be necessary in the future.

## Conclusions

Proteinuria and hematuria are both important pathologic findings, but before the present study, we did not know the relationship between these conditions and AKI or mortality in critically ill patients. The present study cannot determine whether reducing the amount of proteinuria or hematuria decreases the risk of AKI or mortality in the ICU subset. Furthermore, although the dipstick test has the advantage of being inexpensive, the cost-effectiveness of the dipstick test is not assured from the study results. However, the present study will form the basis of later studies to address these issues.

## Competing interest

The authors declare that they have no competing interest.

## Authors’ contributions

SSH designed the study, collected data, analyzed and interpreted the results, and drafted the manuscript. SYA, JWR, and SHB collected data and analyzed the results. HJC participated in acquisition of data and interpreting the results. KYN and DWC designed the study and edited the manuscript. SK conceived the study, analyzed the results, interpreted the data, and reviewed the manuscript. All authors read and approved the final manuscript.

## Pre-publication history

The pre-publication history for this paper can be accessed here:

http://www.biomedcentral.com/1471-2369/15/93/prepub
